# Technology for Blending Recombined Flour: Substitution of Extruded Rice Flour, Quantity of Addition, and Impact on Dough

**DOI:** 10.3390/foods13182929

**Published:** 2024-09-16

**Authors:** Xuyang Ren, Huining Zhang, Mingshou Lv, Hongchen Fan, Linlin Liu, Bing Wang, Xiaofeng Hu, Yanguo Shi, Chunhua Yang, Fenglian Chen, Ying Sun

**Affiliations:** 1 College of Food Engineering, Harbin University of Commerce, Harbin 150028, China; rxyhsd@163.com (X.R.); lemonzhn0923@163.com (H.Z.); lumingshou@hrbcu.edu.cn (M.L.); fanhongchen@hrbcu.edu.cn (H.F.); keaiduolinlin@126.com (L.L.); iceking85@163.com (B.W.); shoho1125@163.com (X.H.); yanguosh@163.com (Y.S.); yangchunhua25925@126.com (C.Y.); 2 College of Tourism and Cuisine, Harbin University of Commerce, Harbin 150028, China

**Keywords:** extrusion, rice flour, dough

## Abstract

In a previous study, rice bread was prepared using a combination of rice–wheat mixed flour. To investigate the impact of the partial adoption of extruded rice flour (ERF) on mixed flour (MF) and mixed dough (MD), the effects of adding ERF on the pasting, mixing characteristics, texture, and water retention of the MF and MD were examined by a rapid visco analyzer (RVA), Mixolab, texture profile analysis (TPA), and a low-field nuclear magnetic resonance analyzer (LF-NMR). The PV, TV, BD, FV, and SV of the MF declined as the incorporated amount of ERF increased. There was no significant difference in the PT at the 5–15% addition level (*p* < 0.05), but it showed an increasing trend at the 20–30% level (*p* < 0.05). The incorporation of ERF led to a significant increase in the water absorption (WA) of the MD, while the DT, ST, C2, C3, C4, and C5 exhibited a declining trend. The texture analysis revealed a significant decrease in the dough hardness with the addition of ERF, with a 55% reduction in the hardness of the 30% improved mixed dough (IMD), and the cohesiveness increased significantly (*p* < 0.05). The IMD was mainly composed of weakly bound water. The content of weakly bound water increased with the ERF amount.

## 1. Introduction

As an important part of the diet, rice has many excellent characteristics and nutritional qualities. The biological potency and amino acid composition ratio of rice protein are higher than those in general cereal crops. Rice protein is a resistant protein, namely, producing no allergic reaction. Therefore, rice is one of the preferred alternatives to wheat, which can cause celiac disease because of its gluten protein (glutenin and gliadin) [1]. Gui et al. [2] found that rice could replace wheat to make gluten-free bread, providing a food choice for celiac patients. However, rice flour is unable to develop the same network structure in the dough as wheat flour because of the absence of gluten, which makes it difficult to form viscoelasticity when kneading. As a result, for rice bread, the volume is small, the texture and taste are poor, and the shelf life is short. Therefore, in recent years, many researchers have carried out relevant studies to control the quality of gluten-free bread by using the addition of hydrocolloid [3,4], protein [5,6,7], chemical acidification [8,9], or extrusion technology [10,11,12].

Extrusion technology involves the crushing, conditioning, blending, and, ultimately, shaping of food raw materials using extrusion equipment [8]. In the high-temperature and pressure environment of the extruder, the rice flour is subjected to mixing, stirring, friction, and shear force, by which the protein is denatured and the starch is pasted. Furthermore, the thermal processing of extrusion results in chemical changes in nutritional and functional properties, such as changes in lipid oxidation, Maillard browning, the inactivation of anti-nutritional compounds, changes in antioxidant properties, changes in bioactive compounds, and the improvement of sensory attributes [13]. Many researchers have used extrusion technology to modify rice, wheat, oats, and other grains and compound raw materials [11,14,15]. It was found that wheat dough with added ERF had a high viscous modulus (G′) and elastic modulus (G′′). Its hardness underwent a significant decrease, while its ductility underwent an increase, which was beneficial to the dough's rheological qualities and the bread quality [16]. Generally, extrusion can lead to varying degrees of gelatinization of the starch in the flour, thereby influencing the quality of the extrudates [17]. Seetapan et al. [18] found that the starch particles in noodles were mostly broken and pasted after the rice flour was extruded, resulting in a great coherent structure and ductility after cooking. Lai et al. [19] studied rice flour extruded with different water contents and found that a high water content could ease digestion by improving the branching of the starch structure and weakening the degradation of the protein. Yan et al. [12] produced gluten-free brown rice bread using extruded rice starch with different moisture contents. It was found that compared with the control group, the dough with extruded rice starch with a 40% water content displayed low hardness, high elasticity, and resistance to deformation. Martínez et al. [20] found that the use of extruded flour in rice bread making could increase the hydration of the dough, thereby increasing the yield of the bread and reducing its aging. However, it remains to be witnessed whether dough formed by extrusion-modified rice–wheat flour possesses favorable properties suitable for rice production. In addition, the majority of studies have merely examined the impacts of extrusion technology on the physicochemical and structural attributes of rice flour and broken rice [19,21,22], but there are scarce reports regarding the effects of the amount of ERF added to the rice–wheat flour on the mixing characteristics and pasting properties of the dough. Thus, the main objective of this study was to explore the effect of ERF on improving the MF and MD. The improved rice flour was obtained by adding ERF to the MF according to a certain amount, and the effect of the ERF on the quality of the MD was studied. As an effective means to modify rice flour, the extrusion technology was further applied to rice products to provide theoretical knowledge about ERF for future research and production.

## 2. Materials and Methods

### 2.1. Materials

The wheat flour (11.05% ± 0.03 protein, 1.13% ± 0.12 fat, 68.74% ± 0.40 starch, 16.10% ± 0.01 amylose, and 11.22% ± 0.07 water) was provided by Shenyang Zhongliang Food Co., Ltd. The rice flour (5.55% ± 0.02 protein, 0.72% ± 0.23 fat, 85.58% ± 0.65 starch, 16.20% ± 0.09 amylose, and 10.10% ± 0.01 water) was provided by Anjia Village, Wuchang City, Heilongjiang Province.

### 2.2. Methods

#### 2.2.1. Preparation of Extrusion-Modified Rice–Wheat Flour Mixed Powder

On the basis of the rice–wheat flour mixture (rice–wheat = 4:6), ERF was added to the rice–wheat flour mixture in the form of ERF instead of the original rice flour, and the substitution amounts were 5, 10, 15, 20, 25, and 30% (according to the quality of the rice–wheat flour mixture substitution) to obtain the modified rice–wheat flour mixtures.

#### 2.2.2. Pasting Properties

A Rapid Visco Analyzer (RVA-TECMASTER, Swedish Botong Company, Sweden) paste analysis was performed according to standard AACC Approved Method 76–21 [23] and modified to determine the effect of the substitution of different extruded puffed rice flours on the pasting characteristics of the mix. Three-point 3.5 ± 0.01 g samples (moisture basis: 14%) and 25 ± 0.1 g of distilled water were weighed into an aluminum canister and tested according to the following procedure: The samples were equilibrated at 50 °C for 60 s, heated to 95 °C at a rate of 5 °C/min for 9 min, and maintained at 95 °C for 7 min. The temperature was reduced to 50 °C at a rate of 6 °C/min and, finally, held at 50 °C for 7.5 min and maintained at 50 °C for 4.5 min. The rotating speed was 960 r/min in the first 10 s and maintained at 160 r/min during the whole process.

#### 2.2.3. Rheological Properties

Mixolab (Mixolab 2, French Chopin Technology Company, Paris, France) was used to study the changes in the mixed flour dough with different substitution amounts of ERF during stirring formation, heating gelatinization, and cooling. The dough weight was set at 75 g. The determination process was as follows: The samples were maintained at 30 °C for 8 min, heated to 90 °C at a rate of 4 °C/min, and maintained at 90 °C for 7 min. The temperature was reduced to 50 °C at a rate of 4 °C/min and maintained at 50 °C for 5 min. The total time was 45 min. The stirring speed was always 80 r/min. Each group of samples was repeated 3 times parallel.

#### 2.2.4. Low-Field Nuclear Magnetic Resonance (LF-NMR)

A previous method described by Li et al. [24] was used with some modifications. The samples (3 g) were transferred into NMR glass tubes. The tests were conducted using the Carr–Purcell–Meiboom–Gill (GPMG) sequence and set as follows: 3000 echoes (C0), 16 scans (NSs), and a TE of 0.15 ms. The CONTIN software was used to call the CPMG sequence inversion, and each peak time constant T_2i_ (peak time) and its area fraction M_2i_ were recorded for subsequent analysis.

#### 2.2.5. Textural Properties

Texture profile analysis (TPA, TA. new plus, Shanghai Unico Instrument Co., Ltd., Shanghai, China) was performed on the mixed flour dough using a texture analyzer, and at least three parallel doughs were made for each group of samples. The length, width, and height of each sample were all set as 30 mm. The probe type [25] was a Phammer 35; the pre-test speed was 1 mm/s; the test speed was 1 mm/s; the post-test speed was 2 mm/s; the trigger force was 5 g; and the strain was 30%.

### 2.3. Statistical Analysis

The Origin 2018 software was used for the analysis and graphing. The SPSS 17.0 software was used to process and analyze the experimental data. Analysis of variance (ANOVA) was performed by Duncan’s tests, and *p* < 0.05 was considered a significant difference.

## 3. Results and Discussion

### 3.1. Pasting Properties

**Figure 1:**
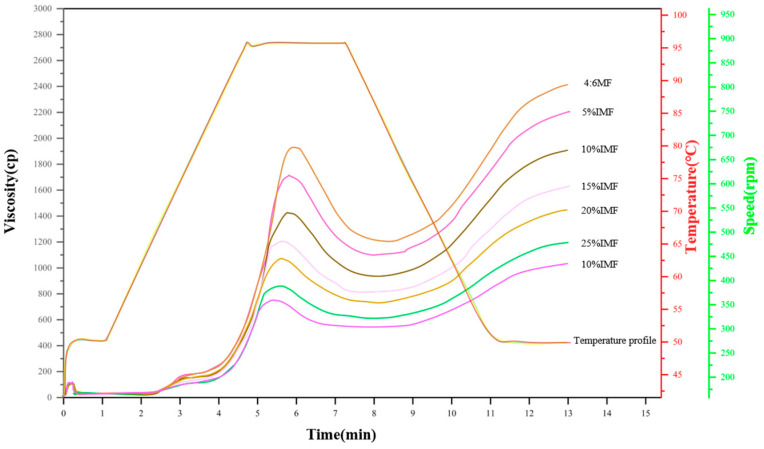
RVA patterns of MF samples with different addition amounts of ERF.

The pasting characteristics are important measurements for starch-derived food. The RVA pasting curves of the improved mixed flour (IMF) samples are shown in Figure 1, and the values of the pasting parameters are shown in Table 1. The pasting parameters of the IMF decreased with the addition of ERF, except for the increasing pasting temperature.

The peak viscosity (PV) represents the swelling degree of starch under heating. The greater the amylopectin content, the higher the PV [26]. The trough viscosity (TV) is the trough viscosity of starch under shear. The degree of crystal ordering and crystallinity decreased after the rice flour was extruded, and the amylopectin was degraded [27]. Moreover, the extrusion process promoted starch gelatinization, resulting in a lower number of natural starch particles [28]. Therefore, with the increasing addition of ERF, the PV and TV showed a decreasing trend. At the 30% addition level, they were reduced to the minimum values of 723.67 and 518.67cp, respectively. Wang et al. [29] observed that the starch content available for gelatinization in bran flour blends decreased after the extrusion of the broken rice and rice bran, leading to a reduction in the PV of the rice flour. This finding aligns with our results.

The breakdown value (BD) represents the resistance to shear and heat of the starch thermal paste. A small BD value means good thermal stability [30]. With the increasing addition of ERF, the BDs of the IMF samples gradually decreased and were all significantly less than that of the MF (*p* < 0.05). The BD achieved the lowest value at a level of 30%, showing the great stability of the thermal paste of the IMF. The possible reason was that the ERF had a higher degree of pasting and hardly any whole starch particles, resulting in a higher concentration of starch molecules in the solution, which increased its thermal stability [31,32].

The final viscosity (FV) represents the aging ability of starch [33]. With the increasing addition of ERF, the FV of the IMF decreased, indicating less aggregation of the starch molecules when cooling. The setback viscosity (SV) reflects the stability and aging ability of the cold starch paste. The lower the SV, the better the stability of the cold paste, and the starch is less susceptible to aging [34]. With the increasing addition of ERF, the SV of the IMF showed a significantly decreasing trend (*p* < 0.05). At the 30% addition level, the SV reached the lowest value of 497.00 cp, indicating that ERF enhanced the aging resistance of the MF. This was probably because, after extrusion, the degree of crystal ordering and crystallinity of the rice starch was reduced, which was unable to provide the base nuclei for aging [27]. These results are consistent with the FV results.

It has been reported that starch with a high content of amylose means high crystallinity, a high-calorie requirement for dissolution, and a higher pasting temperature (PT). Firstly, although the crystallinity of the rice flour was diminished, the high temperature during extrusion caused the unwinding of the double helices and the leaching of amylose. Subsequently, this amylose underwent aging, resulting in an increase in the PT. Secondly, the formation of complexes inhibited the expansion of starch particles and contributed to a rise in the PT [35,36,37]. Amylose readily formed amylose–lipid complexes with fats, further elevating the PT. In addition, it was found that the starch particles in the rice were incomplete and fragmented after extrusion. The particles were squeezed and aggregated to form amorphous flake structures, which should have also contributed to the increase in the PT [29,38].

### 3.2. Thermomechanical Properties

The Mixolab parameters are presented in Table 2. The addition of ERF significantly increased the water absorption (WA), while the values of the dough time (DT), the stabilization time (ST), C2, C3, C4, and C5 were reduced.

The increase in the WA indicated the high water-binding capacity. It demonstrated that the dough with ERF needed to absorb more water compared with the MD. This most likely was attributed to the starch in the rice being pasted and its crystal structure being destroyed after extrusion. Meanwhile, a large number of hydrophilic groups, such as hydroxyl, were exposed, resulting in hydration with water [5], consistent with Martínez et al.’s research [39]. Hagenimana et al. [40] reported that the addition of extruded wheat flour significantly increased the WA but decreased the DT and ST. The large amount of water required for dough formation helped to increase productivity.

The DT of the IMD initially decreased with the addition of ERF and then increased after reaching a minimum of 0.73 min at the 20% addition level. Generally, the DT is related to the protein properties and WA. The extrusion treatment led to the degradation of the protein, resulting in a reduction in its content [5]. When the addition amount was low, the reduced protein content resulted in a decrease in the DT. Furthermore, Jafari et al. [41] showed that extrusion reduced water–protein interactions. Meanwhile, moisture competition between the pasted starch in the ERF and protein delayed dough formation, thus raising the DT when the addition amount was over 20%.

The ST of the IMD initially decreased from 6.10% to 1.20% with the addition of the ERF and then remained at 1.20% at the 15% addition level. A stable strength usually depends on the three-dimensional network structure of the hydrophilic colloid formed by the protein and water during the kneading of dough [42]. Generally, disulfide bonds are critical in determining the quality attributes of the three-dimensional network structure of the dough [43]. It was reported that the free and total sulfhydryl contents of rice protein increased, while the disulfide bond content decreased, during the extrusion process [44]. Meanwhile, the β-turn content increased, but the α-helix content decreased [45]. Generally, the α-helix content of protein secondary structures has an important effect on the hardness and springiness of the three-dimensional structure [46], while the β-turn content has an important effect on its viscosity [47]. Consequently, changes in the secondary structures and bonding of the tertiary structures affected the stability of the dough. In addition, we supposed that the ST should be related to the pre-pasteurized state of the starch in the ERF. After pasting, the starch molecules were released from the granules and hydrated with water to form a viscous hydrogel when kneading, which had a certain influence on the ST of the dough.

The C2 torque is the minimum torque produced when dough is subjected to both temperature and thermomechanical stress and the protein is weakened during the mixing process. The C2 of the IMD decreased by adding ERF, indicative of protein weakening or structural instability [48]. This was probably related to the degradation and denaturation of the proteins in the ERF. A dough is unstable when the C2 is too low; inversely, it becomes excessively hard if the C2 is too high, resulting in a low bread volume [49].

C3 is the maximum torque produced by starch gelatinization. C4 is affected by the α-amylase inherent in wheat flour related to the stability of starch gels. C5 indicates the starch retrogradation in the cooling stage. Generally, C3, C4, and C5, related to starch gelatinization, have an important influence on the bread volume and texture structure [50,51]. If C3, C4, and C5 are reduced, the starch gelatinization ability is reduced, and the gluten strength of the dough is weakened, resulting in a smaller bread volume. With the increase in the ERF amount, the C3 torque, the minimum C4 torque after 90 °C, and the C5 torque after cooling at 50 °C were significantly decreased (*p* < 0.05). These results are consistent with those of the PV, TV, and SV in the RVA.

C3–C4 indicates the stable range of heating treatment, which has been found to be negatively correlated with cooking stability. The smaller the C3–C4 value, the better the stability [52]. The C3–C4 value decreased significantly with the increasing ERF amount (*p* < 0.05), and the smallest value was 0.07% at the 30% addition level, indicating the great stability of the IMD. This was consistent with the BD. There was no significant effect on the C5–C4 value by the addition of the ERF. It has been found that the C5–C4 value is not affected by the degree of cooking or milling [53].

### 3.3. Textural Properties

Hardness is the magnitude of force required to deform an object. The hardness of the IMD was significantly decreased with the increase in the ERF amount (*p* < 0.05). At the 30% addition level, the hardness reached the lowest value of 420gf, reduced by 55% compared with the MD. This may have been due to the increased WA of the IMD after the addition of the ERF, which reduced the hardness [54]. In addition, the protein in the ERF was denatured due to high friction, resulting in a decrease in the dough hardness [55].

The cohesiveness reflects the size of the adhesive force inside the dough. A high cohesiveness indicates a tight bond. As shown in Table 3, the cohesiveness of the IMD increased significantly with the increase in the ERF amount (*p* < 0.05). At the 30% addition level, the cohesiveness reached the maximum value of 1.41gf. After extrusion, the starch in the rice flour was partially gelatinized, resulting in the dissolution of the starch, which increased the number of biomacromolecules in the dough system and the viscosity of the IMD after water absorption. In addition, during the extrusion process of the ERF, a small quantity of free hydroxyl groups was produced, which could interact with amino groups from proteins through hydrogen bonding, thereby enhancing the gel network’s integrity. Consequently, these adjustments modified the internal structure of the hydrophilic gel system within the dough [56]. The macromolecular polymers in the dough increased, and the internal binding force increased; therefore, the cohesiveness increased [57].

Springiness reflects the ability of an object to deform under external forces and return to its original state when the external forces are removed. Resilience represents the ability of the sample to bounce back during the first compression cycle. It is the ratio of the elastic energy released by the returned sample during the first compression cycle to the energy consumption of the probe during compression. As shown in Table 3, with the increase in the ERF level, there was a slight increment in the elasticity and no significant difference in the resilience of the dough (*p* < 0.05). This means that the method was unable to adjust the elasticity of the IMD. The denaturation of the protein in the extruded rice flour was not conducive to the formation of elasticity in the dough, while the dissolution of pregelatinized starch increased the content of biomacromolecules in the dough system, which was conducive to the formation of elasticity. The counteraction of these effects resulted in insignificant changes in elasticity and resilience.

### 3.4. Water-Holding Capacity (WHC) of Dough

**Figure 2:**
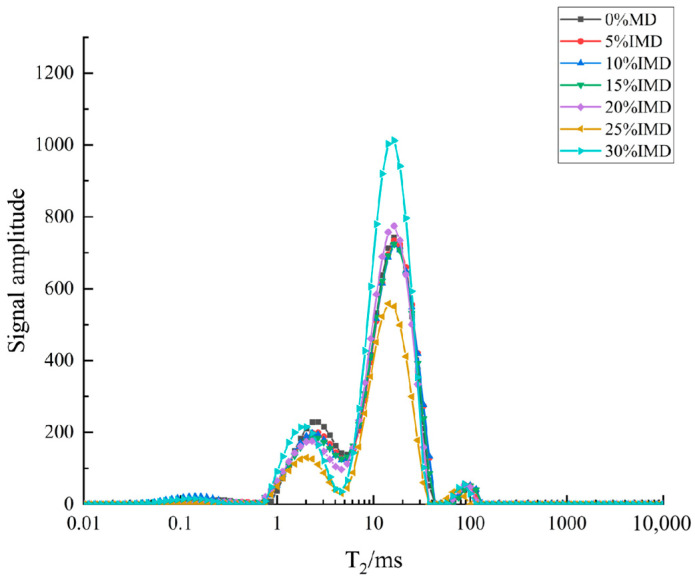
T2 relaxation curves of improved mixed flour dough samples after adding ERF.

The transverse relaxation time T_2_ was used to characterize the water mobility in the dough. The larger the peak T_2_ was, the higher the water freedom was. On the contrary, a small value indicated that the water retention of the dough was strong, and the internal water was closely combined [58]. In the MD of unextruded rice flour, with the development of the dough, the free water formed bound water through hydrogen bonding, and more bound water further bound with the polar groups of the side chains of the protein amino acids [59,60]. With the addition of ERF, the T_2_ shifted to the left, indicating the WHC of the IMD increased. This signified a strong bond between the solids and water in the food [61]. In Figure 2, 2–4 peaks are observed in each curve, indicating that the water in the dough came in different forms. According to the distribution characteristics of the peaks, the water in the dough can be divided into three components: T_21_ (0.1–15 ms), strongly bound water; T_22_ (15–43.29 ms), weakly bound water; and T_23_ (49.77–151.99 ms), free water [62]. 

As shown in Table 4, the addition of ERF decreased the T_21_ of the IMD, but there was no change in the T_22_ (*p* < 0.05). The possible reason was that the denaturation of the protein in the ERF, which reduced the water absorption capacity, resulted in the reduction in the T_21_ and T_23_ [5,39,49]. Secondly, the starch in the ERF was pregelatinized, and the starch particles were broken. With the degradation of amylopectin, a large amount of amylose dissolved, which formed competitive water absorption with the protein and may have also reduced the water content in the network in the protein grid, resulting in the reduction in the T_21_. According to the relative peak area (A_2_), the peak area of A_22_ was the largest, which means that the IMD was mainly dominated by weakly bound water with moderate fluidity. A_23_ was the smallest compared with A_21_ and A_22_, indicating that the dough had the lowest free water content. Compared with the MD, the peak area of strongly bound water decreased by 18%. However, the weakly bound water content increased significantly with the increase in ERF addition (*p* < 0.05). The maximum value reached 81.02% at the 30% addition level, which was 7.34% higher than that of the MD, and the A_23_ increased slightly. This phenomenon should be related to the increase in the starch WA of the ERF. Meanwhile, it may also be that the extrusion treatment reduced the protein in the IMD so that the hydrogen bonds formed by the water binding in the dough were reduced, and the strongly bound water was transformed into weakly bound water and free water [59].

## 4. Conclusions

In this study, adding ERF changed the starch and protein characteristics of the raw materials of wheat and rice flour, which resulted in improving the quality of the MD. It was found that the PV, TV, BD, FV, and SV decreased with the increase in the ERF content. There was no significant difference in the PT with the addition of ERF at 5–15% (*p* < 0.05). However, at the addition level of 20–30%, the PT showed an increasing trend, indicating that the extrusion treatment had a significant effect on the gelatinization characteristics of the raw materials. The Mixolab analysis showed that the WA of the IMF was positively correlated with the amount of ERF added, while the DT, ST, and other torque values were negatively correlated. The textural analysis showed that the addition of ERF significantly reduced the hardness of the MD. However, the cohesiveness increased, indicating that the internal structure was strengthened. There was no significant effect on the springiness or resilience (*p* < 0.05). The IMD was mainly composed of weakly bound water, and the content of weakly bound water increased with the addition of ERF, but the binding degree did not change. However, the content of strongly bound water and its binding degree decreased. In short, the starch pasted and the protein denatured after the extrusion treatment of the rice flour. In the formation of the dough, the WA and combination of the starch and protein, as well as the structure, were changed. The present conclusions may provide a reference for the application of extruded powder in food processing.

## Figures and Tables

**Table 1:** Eigenvalues in the process of improved mixed paste samples.

Sample	PV/cP	TV/cP	BD/cP	FV/cP	SV/cP	PT/°C
MF	1935.67 ± 9.24 ^b^	1204.00 ± 1.73 ^b^	731.67 ± 7.51 ^b^	2413.67 ± 4.04 ^b^	1209.67 ± 2.31 ^b^	88.02 ± 0.03 ^c^
5%IMF	1690.33 ± 9.81 ^c^	1078.67 ± 7.90 ^d^	611.67 ± 8.08 ^c^	2178.67 ± 17.90 ^d^	1100.00 ± 0.01 ^d^	88.47 ± 0.46 ^c^
10%IMF	1394.00 ± 7.32 ^d^	922.00 ± 9.05 ^e^	472.00 ± 1.73 ^d^	1873.00 ± 32.91 ^e^	951.00 ± 13.86 ^e^	88.20 ± 0.52 ^c^
15%IMF	1185.00 ± 5.20 ^e^	800.00 ± 3.46 ^f^	385.00 ± 1.73 ^e^	1616.67 ± 6.35 ^f^	816.67 ± 2.89 ^f^	88.56 ± 0.06 ^b^
20%IMF	1042.33 ± 4.62 ^f^	720.00 ± 1.73 ^g^	322.33 ± 2.89 ^f^	1435.67 ± 5.77 ^g^	715.67 ± 4.04 ^g^	89.63 ± 0.03 ^a^
25%IMF	844.33 ± 2.31 ^g^	594.67 ± 1.15 ^h^	249.67 ± 1.15 ^g^	1178.67 ± 1.51 ^h^	584.00 ± 0.01 ^h^	89.62 ± 0.03 ^a^
30%IMF	723.67 ± 10.97 ^h^	518.67 ± 5.77 ^i^	205.00 ± 5.20 ^g^	1015.67 ± 9.24 ^i^	497.00 ± 3.46 ^i^	89.63 ± 0.06 ^a^

Note: PV, peak viscosity; TV, trough viscosity; BD, breakdown value; FV, final viscosity; SV, setback viscosity; PT, pasting temperature. Each value is represented as the mean ± standard deviation (n = 3). Different letters for the same level of substitution indicate a significant difference (*p* < 0.05). The same letter indicates no significant difference.

**Table 2:** Thermomechanical properties of extruded and expanded improved mixed dough samples.

Mixolab	Sample						
	MD(0%)	IMD5%	IMD10%	IMD15%	IMD20%	IMD25%	IMD30%
WA/%	61.10 ± 0.00 ^d^	61.1 ± 0.01 ^d^	61.2 ± 0.02 ^d^	61.5 ± 0.01 ^c^	61.5 ± 0.02 ^c^	61.4 ± 0.02 ^c^	63.7 ± 0.01 ^b^
DT/min	1.29 ± 0.02 ^a^	0.87 ± 0.00 ^e^	0.88 ± 0.02 ^e^	0.77 ± 0.04 ^f^	0.73 ± 0.01 ^f^	0.85 ± 0.03 ^e^	0.93 ± 0.06 ^d^
ST/min	6.10 ± 0.02 ^b^	4.10 ± 0.04 ^c^	2.80 ± 0.02 ^e^	1.20 ± 0.00 ^f^	1.20 ± 0.01 ^f^	1.20 ± 0.03 ^f^	1.10 ± 0.02 ^f^
C2/Nm	0.40 ± 0.04 ^b^	0.32 ± 0.02 ^c^	0.24 ± 0.00 ^d^	0.20 ± 0.01 ^e^	0.19 ± 0.03 ^e^	0.18 ± 0.00 ^e^	0.15 ± 0.01 ^f^
C3/Nm	1.95 ± 0.01 ^b^	1.82 ± 0.00 ^c^	1.70 ± 0.02 ^d^	1.53 ± 0.00 ^e^	1.39 ± 0.01 ^f^	1.20 ± 0.00 ^g^	0.98 ± 0.01 ^h^
C4/Nm	1.42 ± 0.02 ^b^	1.43 ± 0.00 ^b^	1.38 ± 0.02 ^c^	1.30 ± 0.00 ^e^	1.23 ± 0.00 ^f^	1.10 ± 0.01 ^g^	0.91 ± 0.04 ^h^
C5/Nm	1.98 ± 0.02 ^b^	1.90 ± 0.01 ^d^	1.84 ± 0.00 ^e^	1.77 ± 0.03 ^f^	1.70 ± 0.00 ^g^	1.56 ± 0.01 ^h^	1.28 ± 0.01 ^i^
C3–C4/Nm	0.53 ± 0.01 ^b^	0.39 ± 0.00 ^c^	0.32 ± 0.00 ^d^	0.23 ± 0.00 ^e^	0.16 ± 0.01 ^f^	0.10 ± 0.01 ^g^	0.07 ± 0.03 ^h^
C5–C4/Nm	0.56 ± 0.00 ^c^	0.47 ± 0.01 ^d^	0.46 ± 0.02 ^d^	0.47 ± 0.03 ^d^	0.47 ± 0.00 ^d^	0.46 ± 0.00 ^d^	0.37 ± 0.03 ^e^

Note: Means with different letters within the same parameter differ significantly (*p* < 0.05). C2, protein network strength under increased heating; C3, maximum torque produced during the heating stage (gelatinization); C4, minimum torque reached during heating; C5, torque after cooling to 50 °C; C3–C4, stability during the heating stage; and C5–C4, tendency.

**Table 3:** Influence of ERF on textural characteristics of improved mixed flour dough.

Sample	Hardness/gf	Springiness	Cohesiveness/gf	Resilience
0%MD	1389.33 ± 85.14 ^a^	0.20 ± 0.01 ^c^	1.28 ± 0.01 ^c^	0.10 ± 0.01 ^b^
5%IMD	940.67 ± 34.12 ^b^	0.24 ± 0.03 ^b,c^	1.06 ± 0.01 ^d^	0.10 ± 0.01 ^b^
10%IMD	811.33 ± 52.32 ^c^	0.23 ± 0.02 ^b,c^	1.26 ± 0.03 ^c^	0.09 ± 0.01 ^b^
15%IMD	662.67 ± 39.00 ^d^	0.26 ± 0.04 ^b^	1.30 ± 0.01 ^b^	0.10 ± 0.01 ^b^
20%IMD	477.33 ± 44.74 ^e^	0.23 ± 0.03 ^b,c^	1.32 ± 0.12 ^b^	0.10 ± 0.01 ^b^
25%IMD	433.33 ± 37.22 ^e^	0.23 ± 0.03 ^b,c^	1.31 ± 0.12 ^b^	0.10 ± 0.01 ^b^
30%IMD	420.57 ± 23.58 ^e^	0.22 ± 0.01 ^b,c^	1.41 ± 0.07 ^a^	0.10 ± 0.01 ^b^

Note: Means with different letters within the same parameter differ significantly (*p* < 0.05).

**Table 4:** Influence of ERF on moisture distribution of improved mixed flour dough samples.

	Relaxation Time (T_2_)/ms			Relative Peak Area (A_2_)/%		
	T_21_	T_22_	T_23_	A_21_	A_22_	A_23_
0%MD	2.66 ± 0.01 ^a^	16.30 ± 0.15 ^a^	100.00 ± 1.30 ^b^	24.55 ± 0.18 ^a^	73.68 ± 0.77 ^d^	1.78 ± 0.01 ^c^
5%IMD	2.66 ± 0.04 ^a^	16.30 ± 0.32 ^a^	91.97 ± 2.00 ^c^	23.07 ± 0.14 ^b^	74.81 ± 0.10 ^d^	2.12 ± 0.02 ^a^
10%IMD	2.31 ± 0.01 ^b^	16.30 ± 0.14 ^a^	100.00 ± 1.22 ^b^	21.03 ± 0.11 ^c^	76.94 ± 0.21 ^c^	2.03 ± 0.00 ^b^
15%IMD	2.31 ± 0.02 ^b^	16.30 ± 0.22 ^a^	100.00 ± 1.34 ^b^	21.04 ± 0.13 ^c^	77.15 ± 0.18 ^c^	1.81 ± 0.02 ^c^
20%IMD	2.31 ± 0.03 ^b^	16.30 ± 0.16 ^a^	86.97 ± 1.23 ^c^	19.76 ± 0.16 ^d^	78.28 ± 0.17 ^b^	1.97 ± 0.01 ^b^
25%IMD	2.01 ± 0.01 ^c^	16.30 ± 0.21 ^a^	86.97 ± 1.22 ^c^	19.11 ± 0.11 ^d^	78.86 ± 0.12 ^b^	2.03 ± 0.03 ^b^
30%IMD	2.01 ± 0.02 ^c^	16.30 ± 0.20 ^a^	86.97 ± 1.34 ^c^	17.39 ± 0.12 ^e^	81.02 ± 0.14 ^a^	1.99 ± 0.01 ^d^

Note: Means with different letters within the same parameter differ significantly (*p* < 0.05).

## Data Availability

The original contributions presented in the study are included in the article further inquiries can be directed to the corresponding authors.
